# Prevalence and distribution of the *stx_1_*, *stx_2_* genes in Shiga toxin producing *E. coli* (STEC) isolates from cattle

**Published:** 2010-03

**Authors:** Y Tahamtan, M Hayati, MM Namavari

**Affiliations:** Razi Vaccine and Serum Research institute Shiraz-Iran

**Keywords:** STEC, stx1, stx2, cattle, Iran

## Abstract

**Background and Objectives:**

Shiga toxin-producing *Escherichia coli* (STEC) strains are human pathogens linked to hemorrhagic colitis and hemolytic uremic syndrome. Shiga toxins (Stx1 and Stx2) are the major virulence factors of these strains. The aim of this work was to study the prevalence and distribution of *stx*_*1*_ and *stx*_*2*_ gene in *E. coli* O157:H7 and non-O157:H7 strains isolated from cattle in Shiraz, Iran.

**Materials and Methods:**

Four hundred and twenty samples consisted of recto-anal mucosal swabs were collected from cattle. They were checked for the presence of the *stx*1 and *stx2* gene using multiplex-PCR every 1 week over a 1-year period (2007-2008).

**Results:**

A total of 146 strains carrying the *stx*1 and *stx2* gene were isolated from 51 (12.14%) cattle. Overall, 15 (3.57%) were identified as O157:H7 and 131 (31.19%) revealed to be non-O157:H7. Both *stx2* and *stx*1 genes were detected in 51 (34.93%) STEC isolates. Genotypes *stx*1 and *stx2* were detected in 15 (10.27%) and 78 (53.42%) respectively. Seasonal distribution of *stx* genes revealed high percentage of positive animals in warm seasons. The gene sequence similarity ranged from 94 to 100%.

**Conclusion:**

Frequency of *stx*1 and *stx2* in animals and its relation to human disease is not well understood in Iran. The high prevalence of STEC in cattle seems to parallel that which is usually observed in warm seasons and it also parallels occurrence of human STEC. The higher prevalence of the *stx2* gene than *stx*1 in strain populations isolated from cattle indicates a risk alert of *E. coli* O157:H7 being shed by cattle in these populations. Appropriate measures are now needed to prevent the spread of this life-threatening foodborne disease in our country.

## INTRODUCTION

The broad group of *E. coli* are known as Enterohemorrhagic *Escherichia coli* (EHEC), including *E. coli* O157:H7 and non-O157 (1). EHEC refers to a subset of Shiga toxin-producing *Escherichia coli* (STEC) strains found to cause human and sometimes animal disease (2). They are linked to development of hemorrhagic colitis (HC), hemolytic uremic syndrome (HUS) (3) and thrombotic hospitalization and intensive care (4). STEC O157:H7 strains were first isolated from cattle in Argentina in 1977, although the strains were identified as such 10 years later (5).

Cattle are considered the primary reservoir of both O157:H7 and non-O157 STEC bacteria (2). Cattle frequently excrete the bacteria in their feces (6). The illness is often linked to the consumption of contaminated and undercooked ground beef. Although other means of transmission (7, 8) have been reported (8).

Several virulence factors have gained importance for the pathogenesis of the STEC infections (9). However, the complete list of bacterial virulence determinants necessary for STEC to cause EHEC-related disease is not known. Shiga toxin is the key factor in STEC pathogenesis (2, 10). Shiga toxin is toxic to human colonic, ileal epithelial (11) and endothelial cells (12). Two main groups of Shiga toxins are harbored in STEC (13–15). Shiga toxin 1 (16) is 98% homologous to the Stx produced by *Shigella dysenteriae* type 1, while Stx2 is about 60% homologous with Stx1 and is antigenically different (9, 17).

*E. coli* O157:H7 is a common cause of bloody diarrhea in developed countries, but its incidence in developing countries including Iran is not clear. The limited prevalence data in foods and animals in Iran has made the assessment of risks difficult, and also the options for management and control are unclear. In Iran, only a few studies have reported the isolation and characterization of STEC in humans (18–20). *E. coli* O157:H7/ H- was sporadic reported to be present in up to 11.5% of cattle in Iran (21). Unfortunately, there is no conclusive data from Middle-East particularly from Iran. Therefore, the objective of this study was to determine the frequency of bacterial virulence profiles of stx in STEC O157 and non-157 in cattle in a one-year period.

## MATERIALS AND METHODS

**Sample collection.** In one year, from September 2007 to August 2008, 420 samples consisting of recto-anal mucosal swabs from cattle were collected in Shiraz-Iran. All samples were collected aseptically, placed in coolers, transported to the laboratory, and kept at 4°C. The samples were immediately processed upon arrival to the laboratory. A questionnaire was carried out for risk factor of *E. coli* O157:H7 determination that included season of the year. The prevalence ratio (PR) was determined using Win Episcope 2.0 at 95.0% level of confidence for association between risk factors.

Seasonal variations in the presence of the stx genes in bacterial populations from rectum were studied by sampling weekly over a 1-year period. For groups of cattle derived from one herd, a random selection of 10% of the total number of cattle was sampled, with a maximum of 10 cattle being sampled. The enumeration of stx gene-carrying bacteria was performed by the MPN-PCR method (Most Probable Number).

**Bacterial strains and media.** *E. coli* O157:H7 strain EDL933 (kindly from Prof. David Gally, University of Edinburgh, UK), which produces Stx1 and Stx2, was used as positive control. *E. coli* O157:H7 strain SHT87 (isolated from cattle in Shiraz-Iran, unpublished) which do not possess the genes stx_1_ and stx_2_, was used as negative control. The bacteria were grown in modified tryptic soy broth and on sorbitol MacConkey agar (supplementation with ceffexime and potassium tellorite) (mTSB, SMAC -CT) (Difco, Le Pont de Claix, France) and incubated at 37°C overnight.

**Isolation and identification of *E. coli.*** All samples were placed in 15 ml of mTSB and incubated overnight at 37°C. The suspension was thoroughly mixed and allowed to stand for a short period before plating. One loop full of enriched broth was streaked onto SMAC-CT. All agar plates were incubated at 37°C for 24 h. Five to six sorbitol negative colonies per sample were collected and streaked on SMAC again. Finally, the bacteria were streaked onto eosin methylene blue (EMB) agar plates and were incubated same as above. The typical *E. coli* metallic shine on EMB were characterized by biochemical tests, including conventional indol, methyl red, voges proskauer, citrate and lysine decarboxylase tests. The identity of *E. coli* O157:H7 was confirmed using an anti-*E. coli* O157 and H7 antisera agglutination kit (Oxoid DR620).

**Nucleic acid isolation.** One ml of overnight mTSB culture from all the bacterial strains was employed as template for PCR. Cells were pelleted from the cultures at 3,000 rpm for 5 min (Hermle Z23o MA centrifuge) and then continued by DNP^TM^ Sina-gene kit (Cat No.: 8115C). All isolates were examined for verotoxin virulence genes determinants by PCR.

**PCR assay.** DNA samples (1µg nucleic acids) were amplified in a 25µl volumes reaction mixture of the following constitution: 2 mM magnesium chloride, 2.5mM for each of dATP, dCTP, dGTP, and dTTP, 2 pM for each of the STX-specific oligonucleotide primers (Oligonucleotide Synthesis Laboratory, Roche, Germany) described in table 1, 1.25 U of *Taq* polymerase (Fermantas, Sylvius, Lithuania) and the final volume was adjusted with sterile double-distilled water.

**Table 1:** Specific oligonucleotide primers used for amplification of stx1 and stx2 gene.

Oligonucleotides	Size	Reference
Stx1 F CTT CGG TAT CCT ATT CCC GG	484	(35)
Stx1 R GGA TGC ATC TCT GGT CAT TG		
		
Stx2 F CCA TGA CAA CGG ACA GCA GTT	779	
Stx2 R CCT GTC AAC TGA GCA GCA CTT TG		

The samples were overlaid with 100 µl of mineral oil, denatured at 94°C for 5 min, and subjected to 30 cycles of amplification in a DNA Thermal Cycler (Ependorf Mastercycler Gradient). Parameters for the amplification cycles were: denaturation for 30 s at 94°C, annealing of primers for 30 s at 56°C, and primer extension for 30 s at 72°C with auto- extension. After the last cycle, the PCR tubes were incubated for 10 min at 72°C. Six microliters of the reaction mixture was then analyzed by standard submarine gel electrophoresis (1.5% agarose; 5 V/cm), and the reaction products were visualized by being stained with ethidium bromide (0.5, µg/ml in the running buffer).

Bromocresol broth tubes containing 1% D-sorbitol (Sigma, St. Louis, Mo.) were used for the sorbitol fermentation test. All susceptible strains were separately incubated in 250 µl of phosphate-buffered saline with β -glucuronidase tablet (Diatabs, Rosko, Denmark).The incubation time and temperature was 24h at 37°C.

**Sequencing:** The strains harboring shiga toxin were sequenced both in reverse and forward with the same primers used for *stx* genes. The obtained sequences were balsted in NCBI databases. The data was analyzed with SPSS (Statistical Package for Social Sciences) for Windows version 11.5 software with assumed confidence level of 95%.

## RESULTS

The majorities of the isolated strains were not able to ferment sorbitol within 24 h and had β-D-glucuronidase activity. Only ten strains were able to ferment sorbitol within 24 h, of which only one belonged to the *E. coli* O157: H^_^ serotype. This serotype was also negative for the β-D-glucuronidase test. The data point to the high prevalence of stx_2_ in our study both in O157 and non-O157.

All animals came to the Shiraz slaughterhouse from farms located in different regions of the state. No significant difference in STEC isolation rate was observed when the cattle were grouped according to their geographical origin.

**Isolation and characterization of STEC in cattle.** A total of 146/420 (34.76%, 95% CI) STEC strains were isolated from 51 (12.14%) out of the 420 cattle, that posses *stx*_*1*_ and/or *stx*_*2*_. Fifteen (3.57%) isolates were classified as *E. coli* O157:H7 and 131(31.19%) non-O157 (Table 2).

**Table 2:** Distribution of *E. coli* O157, Non-O157 stx1, stx2 Genes in Cattle. Shiraz-2007-8.

Strains (No.)	Stx1	Stx2	Stx1+stx2	-stx	Total%
**O157: H7**	6	8	6	6	15(3.57)
**Non- O157**	60	121	45	15	131(31.19)
**Total**	66	129	51	21	146(34.76)

**Isolation and characterization of stx gene-carrying STEC bacteria.** Any *E. coli* isolated harboring at least one shiga toxin gene was considered positive for STEC. Both *stx*_*2*_ and *stx*_*1*_ genes were detected in 51(34.93%) isolates, but *stx*_*1*_ was detected in 15 (10.27%) and *stx*_*2*_ was detected in 78 (53.42%). One or more cattle from each Shiraz farm was positive for *stx*. The ratio of *stx*_*2*_ to *stx*_*1*_ gene-carrying bacteria was 5.2:1. Except for the six strains that apparently lost the genes, the presence of the *stx* gene was confirmed by specific PCR for all of these isolates.

**Fig. 1:**
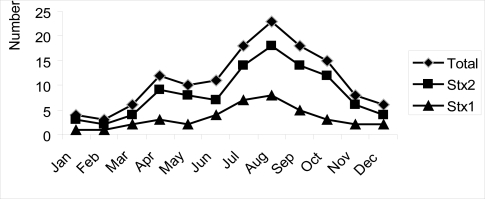
Seasonality STXs from STEC isolates from cattle Shiraz-Iran.2007–8

**Seasonal distribution of the** ***stx*** **genes.** The proportion of each of these bacterial populations that carried *stx*_*2*_ and/or *stx*_1_ were not similar (Fig. 1). There was a significance seasonal difference for any of the measured parameters, as indicated by an analysis of variance test (P< 0.05). The percentage of positive animals range from 24.28 to 40.9% in warm seasons of May to August (in Iran) compared to winter seasons with the average frequency dropping (8.96 to 11.11%).

**Sequencing.** Some of the 484 and 779 bp (Table 1) amplimers were sequenced. These phages were isolated from strains summarized in Table 3.

**Table 3:** Sequence comparison of stx2 genes in different strains.

Identity to stx1,2 gene of EDL933 (AE005174.2)%			
**Strains ( Stx1)**	Identity to stx1	Identity to stx2	Strains ( Stx2)

**DEC10J, EC108**	100	98	c466-01B
**EC127, EC120**	99	98.2	EC130
**BCN26**	94	98.1	EC176, EC125
**EC152**	99	98.6	EC169, EC131
**EC176**	99	97.9	H2687 serotype O157:NM
**O157:H7 Str. Sakai**	99	99	I8257, 933W slt-II
**AB8SF, ECLR2**	91	99.1	O157:H7 str. Sakai

## DISCUSSION

This is the first study which describes the detection and frequency of major virulence genes of STEC isolated from cattle in Shiraz, Iran. Our data revealed high levels of stx2 gene-carrying bacteria in fecal samples from different cattle. STEC harboring *stx*_2_ was isolated significantly more (53.42%) than STEC *stx*_1_ (10.27%) (P <0. 01). Most human epidemiological studies in Iran have revealed that the prevalence of STEC infection ranges between 0.7 to 15%, but none of them belonged to the O157:H7 serotype (18, 20). Isolation of STEC from bovine reservoirs from other parts of the country has already been documented (8, 21–24). Zahraee Salehi and his colleagues identified STEC O157 among 7 isolates (11.5%), from cattle, whereas non-O157 strains that are frequently associated with sporadic cases of HUS (25, 26), were isolated from 4 (6%) of animals. They showed 5 (8.2%) isolates carried *stx* genes (21). This finding was in parallel with presence of *stx*_1_ in 35.5 and *stx*_2_ in 49.1% of human isolates (19). This is in contrast with Askari *et al*. finding with a report of *stx*_1_ and *stx*_2_, among 5% and 1.9% of calves respectively (23). Recently Ludwig Kerstin *et al*. reported that 71% of children with HUS were due to Stx2-producing *E. coli* strains (16). In a study in the USA, *stx*_1_ gene was not detected in any strains tested while 93.1% of the isolates were found to carry the *stx*_2_ gene (27).

The gene belonging to strains detected from animals showed more expression of protein toxin than human samples (28), hence the strain within animal origin maintain the characteristic and are more cytotoxic than the gene from human origin (16). This supports the suggestion that cattle may have been the source of the organism for the HUS patients.

The seasonal shedding of STEC and distribution of stx ranged from 8.69 to 40.9%. There are significant seasonal differences in the levels of shedding of stx gene-carrying bacteria. The results revealed decrease in the number of stx gene carrying bacteria during the winter. These results are in agreement with those studies indicating that STEC shedding has seasonal variations by cattle in different countries (5). High isolation rate was observed in late spring and summer in the UK (29), Sweden, Washington State in the USA (30) and Italy (5). In this study, we observed a marked decrease in the prevalence of STEC from October, which in Iran is the beginning of fall, to the end of spring (June). This trend seems to parallel what is usually observed in the summer for the occurrence of human STEC, with very few episodes notified during the winter season (31).

Sequence variations in *stx*_1_ and *stx*_2_ genes of fifteen *E. coli* O157 isolates in this study were investigated. The similarity ranged from 94 to 100%. As demonstrated in the present investigation, the genetic diversity of organisms causing disease is considerable. (GenBank accession number DQ235775).

In conclusion, there is no data available about the frequency of *Stx2* and Stx1 in animal and people in close contact to HUS patients in Iran. The greater observation of the stx2 gene relative to the *stx*_1_ gene in strain populations indicates a risk alert of this gene between these populations (32). Some studies have revealed that strains possessing only *stx*_2_ are potentially more virulent than strains harboring *stx*_1_ or even strains carrying both *stx*_1_ and *stx*_2_ (17, 33). It is of note that most HUS-associated clinically relevant STEC isolates produce Stx2, but at least in Europe, Stx1 is rarely highly relevant (32). Stx2 has been found to be approximately 400 times more toxic (as quantified by LD50 in mice) than Stx1(4).

These findings are important for public health and preventive veterinary medicine. Therefore, emergency cautions are necessary to decrease the incidence of STEC infections in animals and people. In order to achieve this, good hygienic practice and HACCP systems are necessary from the farm to the family table especially in the abattoirs to prevent contamination of meat and abattoir environment with intestinal content.
